# Supplementary material for: Evidence-Based Pharmaceutical Care in Community Pharmacies: A Survey of 595 French Pharmacists
Source: Pharmacy (Basel). 2023 Oct 7;11(5):161. doi: 10.3390/pharmacy11050161 (PMC10610225; doi:10.3390/pharmacy11050161)
Supplement: Supplementary file 1 [file pharmacy-11-00161-s001.zip › S1_Questionnaire_Community pharmacists and their daily therapeutic choices 20231006.pdf]

Supplementary Material S1

Community pharmacists and their daily therapeutic choices

(Translation of original questionnaire in French that was disseminated to French community pharmacists)

This questionnaire is composed of the following 3 parts:

A - 4 clinical situations with proposed multiple-choice answers. There is no right or wrong answer, it's up to you to choose the answer that comes closest to what you would do in your daily practice.

B – 3 questions, concerning your practices at the pharmacy, and your continuing education.

C – Demographics and characteristics of the pharmacy where you work with an optional comment section for any remarks that you would like to make.

This questionnaire is completely anonymous and we assure you that the data will be treated confidentially. It should take only 5 to 10 minutes to complete. Please answer the questionnaire only once per pharmacist.

*Answering these questions is obligatory

Part A

What would you do in the following situations?

- Case n°1

Ms. W, 62 years old, with no major medical history, is coming to get her flu vaccine in October. She says she had a fever for 24 hours following the vaccination last year and has heard that the usual vaccine can be replaced by a homeopathic flu vaccine. What do you do?
Choose one answer:*

☐ I give her the homeopathic product "Influenzinum" instead of the injectable vaccine. I think it is a good option to replace the flu vaccination for this patient.

☐ I advise the patient to try the injectable vaccine first. I think that using homeopathy instead of the vaccine is not a good option to prevent the flu in this patient.

☐ I give her the homeopathic product "Influenzinum" instead of the injectable vaccine. I think that the patient would be better off using the injectable vaccine, but it is difficult for me to discuss this at the pharmacy counter (lack of time, difficulties in going against the patient's wish, economic risk of missing a sale etc.).

☐ I don't know what to do, I will have to look for information before responding to this patient.

• <u>Case n°2</u>

Ms. X, 26 years old, has recently suffered from mild depression diagnosed by her doctor. After discussing different treatment options with her doctor, she wants to try St. John's Wort herbal medicine (Hypericum Perforatum) and asks for your opinion on the matter. She has no other major medical history and does not take any regular treatments or oral contraception. What should you do?
Choose one answer:*

☐ I give him capsules of *Hypericum Perforatum*, from a controlled laboratory. Indeed, I think it is a good option to treat mild depression in this patient.

☐ I advise the patient to use SSRI-type antidepressants instead. Indeed, I think *Hypericum Perforatum* is not a good option for treating mild depression in this patient.

☐ I give him capsules of Hypericum Perforatum, from a controlled laboratory. I think that the patient would be better off using SSRI-type antidepressants, but it is difficult for me to argue for this at the pharmacy counter (lack of time, difficulties in going against the patient's wish, economic risk to miss a sale... etc).

☐ I don't know what to do, I will have to look for information before responding to this patient.

• <u>Case n°3</u>

Mr Y, 55 years old, with no major medical history, is overweight and has high LDL-Cholesterol (> 2g/L). His doctor introduced him to treatment with Atorvastatin 10mg, with the aim of preventing a first cardiovascular accident. Unfortunately, Mr Y tolerated the statin poorly: he quickly complained of muscle pain, which ceased within a few days of stopping the treatment. He now comes to the pharmacy and asks you for some Red Yeast Rice to lower his cholesterol. What do you do?
Choose one answer:*

☐ In addition to offering lifestyle and dietary advice, I deliver Red Rice Yeast. I think it's a good option to decrease the risk of a cardiovascular event for this patient.

☐ In addition to offering lifestyle and dietary advice, I advise the patient not to use this phytotherapeutic product. I think Red Rice Yeast is not a good option to prevent a cardiovascular event in this case.

☐ In addition to offering good lifestyle and dietary advice, I deliver Red Yeast Rice. I think the patient would be better off not using it, but it is difficult for me to discuss this at the pharmacy counter (lack of time, difficulties in going against the patient's wish, economic risk of missing a sale, etc.).

☐ I don't know what to do, I will have to look for information before responding to this patient.

• Case n°4

Mr Z, 38 years old, has suffered from osteoarthritis for several years. As he has no other concurrent pathologies, his doctor prescribed him an oral anti-inflammatory to take during painful episodes. To avoid the adverse cardiovascular effects of NSAIDs as much as possible (increased risk of stroke, heart attack, etc.), you think it is preferable to use:
Choose one answer:*

□ Diclofenac (Voltarene® or other)

□ Naproxen (Apranax® or other)

□ Celecoxib (Celebrex® or other)

□ Ketoprofen (Profenid® or other)

□ I don't know which of these anti-inflammatories causes the least cardiovascular adverse effects.

Part B

My sources of information

- Cases n°1, n°2 and n°3

When dispensing for cases n°1, n°2 and n°3, I have to look for information on homeopathic and phytotherapeutic products, and available OTC. In my usual practice, which resource(s) do I use to find information on this type of product?
For each of the proposed resources listed below please indicate:*

	Never	Infrequently (between 0 and 25% of times)	Quite often (between 26 and 50% of times)	Often (between 51 and 75% of times)	Very often (between 76 and 100% of the times)
SPC or product manual, Vidal, Thériaque etc.	☐	☐	☐	☐	☐
Training materials provided by representatives of pharmaceutical laboratories	☐	☐	☐	☐	☐
Abstracts or full reports and reviews of clinical trials available through search engines such as Pubmed (MEDLINE database), Cochrane reviews or the clinical decision support resource “UptoDate”	☐	☐	☐	☐	☐
Personal experience (personal use of the product, opinions given by patients/customers, family or friends etc.)	☐	☐	☐	☐	☐
Magazines for pharmacists (e.g., Le Moniteur des Pharmacies, Le Quotidien du Pharmacien)	☐	☐	☐	☐	☐
The independent review publication "Prescrire"	☐	☐	☐	☐	☐
Recommendations from health agencies (ANSM, HAS, etc.)	☐	☐	☐	☐	☐
Handbooks for both pharmacists and the public (e.g.: "Advice at the pharmacy pocketbook", "Advice in homeopathy")	☐	☐	☐	☐	☐
Recommendations from learned societies (e.g., SPILF, CRAT, etc.)	☐	☐	☐	☐	☐

Discussion with colleagues (pharmacists, doctors, specialists etc.)	☐	☐	☐	☐	☐

- Case n°4

When dispensing to the patient in case 4, I have to look for information on the prescription of an anti-inflammatory medication, a prescription-only product. In my usual practice, which resource(s) would I use to find information on this type of product?
For each of the proposed resources listed below please indicate:*

	Never	Infrequently (between 0 and 25% of times)	Quite often (between 26 and 50% of times)	Often (between 51 and 75% of times)	Very often (between 76 and 100% of the times)
SPC or product manual, Vidal, Thériaque etc.	☐	☐	☐	☐	☐
Training materials provided by representatives of pharmaceutical laboratories	☐	☐	☐	☐	☐
Abstracts or full reports and reviews of clinical trials available through search engines such as Pubmed (MEDLINE database), Cochrane reviews or the clinical decision support resource “UptoDate”	☐	☐	☐	☐	☐
Personal experience (personal use of the product, opinions given by patients/customers, family or friends etc.)	☐	☐	☐	☐	☐
Magazines for pharmacists (e.g. Le Moniteur des Pharmacies, Le Quotidien du Pharmacien)	☐	☐	☐	☐	☐
The independent review "Prescrire"	☐	☐	☐	☐	☐
Recommendations from health agencies (ANSM, HAS, etc.)	☐	☐	☐	☐	☐
Handbooks available in the dispensary (e.g.: "Advice at the pharmacy in your pocket", "Advice in homeopathy")	☐	☐	☐	☐	☐
Recommendations from learned societies (e.g., SPILF, CRAT, etc.)	☐	☐	☐	☐	☐
Discussion with colleagues (pharmacists, doctors, specialists etc.)	☐	☐	☐	☐	☐

The choice of OTC products

In the following situation: I decide to manage a pharmacy and I have to choose which OTC products I should stock. To choose these products, which arguments seem to me the most/least important in the list below?
Please list as most or least important*

	The product has efficacy demonstrated by studies	The product is frequently requested by patients	The laboratory I work with offers me an interesting market for this product
Argument the most important	☐	☐	☐
Argument the least important	☐	☐	☐

Continuing Education

My continuing education is made through:
You can choose multiple answers. You can add a proposal if needed*

☐ Regular contact with other healthcare professionals (peer group, home/health centre, etc.)
☐ Training material offered by the pharmaceutical laboratories
☐ Attending conferences and training offered by organizations specializing in continuing education (e.g., UTIP, etc.)
☐ Continuing university education (University diplomas in various domains)
☐ Professional magazines for pharmacists (e.g. Le Moniteur des Pharmacies, Le Quotidien du Pharmacien)
☐ The French review journal *Prescrire*
☐ I don't really have any continuing education
☐ Other:

Part C

Please tick or fill in:

Demographic data

Sex:*

☐ Male
☐ Female

Age*

______

Your status in the pharmacy*

☐ Pharmacy owner
☐ Salaried pharmacist (indefinate length contract)
☐ Substitute or temporary pharmacist (fixed term contract)
☐ Junior pharmacist or final year pharmacy student

Year of graduation as a pharmacist*

______

County number of university where you graduated, or country if not in France*

______

Postal code of pharmacy where you work*

______

What university degree/ diploma courses have you followed?*

You can tick multiple answers. You can add other courses if needed.

☐ Orthopedics
☐ Homeopathy
☐ Phytotherapy
☐ Aromatherapy
☐ Nutrition and dietetics
☐ None
☐ Other:

Location of the community pharmacy where you work*

☐ City centre (high population density, strong competition from other pharmacies
☐ Urban area (average population density, limited competition from other pharmacies)
☐ Suburban (peripheral) district with a mostly retired population
☐ Social housing estate
☐ Rural area (small town, village)

Number of qualified pharmacists in the pharmacy where you work:*

______

Number of pharmacy assistants:*

______

Is your pharmacy/dispensary part of a health centre?*
☐ Yes
☐ No

Is your pharmacy part of a chain of pharmacies/chemists?*
☐ Yes
☐ No

Is your pharmacy approved by your reference School of Pharmacy to take pharmacy students on internships?*
☐ Yes
☐ No

Do you have any comments?

Thank you for answering this questionnaire! If you wish, you can comment freely on the subject in the text box below, we will read all your comments with interest. Do not forget to click on "Send" to validate your answers.

____
