# Supplementary material for: Evidence-Based Pharmaceutical Care in Community Pharmacies: A Survey of 595 French Pharmacists
Source: Pharmacy (Basel). 2023 Oct 7;11(5):161. doi: 10.3390/pharmacy11050161 (PMC10610225; doi:10.3390/pharmacy11050161)
Supplement: Supplementary file 1 [file pharmacy-11-00161-s001.zip › Supplementary Tables_ 20231006.pdf]

Evidence Based Pharmaceutical Care in community pharmacies: a survey of 595 French pharmacists.

Supplementary Material

Table S2: Evidence Based Practice Scoring of Multiple-choice answers

Case 1	Score	Comment
1/ I give her the homeopathic product "Influenzinum" instead of the injectable vaccine. I think it is a good option to replace the flu vaccination for this patient.	1	Several meta-analyses conclude that homeopathy is ineffective in preventing influenza (see references in main text 20–22)
/ I advise the patient to try the injectable vaccine first. I think that using homeopathy instead of the vaccine is not a good option to prevent the flu in this patient.	4	
3/ I give her the homeopathic product "Influenzinum" instead of the injectable vaccine. I think that the patient would be better off using the injectable vaccine, but it is difficult for me to discuss this at the pharmacy counter (lack of time, difficulties in going against the patient's wish, economic risk of missing a sale etc.).	3	
4/ I don't know what to do, I will have to look for information before responding	2	
**Case 2***	*	
1/ I give him capsules of *Hypericum Perforatum*, from a controlled laboratory. Indeed, I think it is a good option to treat mild depression in this patient.		For this particular case, all the answers were correct. According to several meta analyzes (23,24) there is no difference between St. John's wort or SSRIs for mild to moderate depression. La Revue Prescrire suggests accepting "prudent use of St. John's wort in case of mild depression, apart from contraindications (children, pregnancy, breastfeeding women, un-tested drug combinations). (25)
2/ I advise the patient to use SSRI-type antidepressants instead. Indeed, I think *Hypericum Perforatum* is not a good option for treating mild depression in this patient.		
3/ I give him capsules of Hypericum Perforatum, from a controlled laboratory. I think that the patient would be better off using SSRI-type antidepressants, but it is difficult for me to argue for this at the pharmacy counter (lack of time, difficulties in going against the patient's wish, economic risk to miss a sale... etc).		
4/ I don't know what to do, I will have to look for information before responding to this patient.		
**Case 3**		
1/ In addition to offering lifestyle and dietary advice, I deliver Red Rice Yeast. I think it's a good option to decrease the risk of a cardiovascular event for this patient.	1	Based on references (26–29)
2/ In addition to offering lifestyle and dietary advice, I advise the patient not to use this phytotherapeutic	4	

product. I think Red Rice Yeast is not a good option to prevent a cardiovascular event in this case.		
3/ In addition to offering good lifestyle and dietary advice, I deliver Red Yeast Rice. I think the patient would be better off not using it, but it is difficult for me to discuss this at the pharmacy counter (lack of time, difficulties in going against the patient's wish, economic risk of missing a sale, etc.).	3	
4/ I don't know what to do, I will have to look for information before responding to this patient.	2	
**Case 4**		
1/ Diclofenac (Voltarene® or other)	1	Based on references (30,31)
2/ Naproxen (Apranax® or other)	4	
3/ Celecoxib (Celebrex® or other)	1	
4/ Ketoprofen (Profenid® or other)	1	
5/ I don't know which of these anti-inflammatories causes the least cardiovascular adverse effects.	2	

*Not included in scoring

20. Prescrire. Homéopathie : toujours pas de preuve d'éfficacité. [Homeopathy: still no proof of effectiveness] Rev Prescrire. 2012;32(344):446. Available at https://www.prscrire.org/Fr/3/31/47896/0/NewsDetails.aspx
21. Ernst E. Homeopathy: what does the "best" evidence tell us? Med J Aust. 2010;192(8):458-60.
22. Demicheli V, Jefferson T, Di Pietrantonj C, Ferroni E, Thorning S, Thomas R, et al. Vaccines for preventing seasonal influenza and its complications in people aged 65 or older | Cochrane. Cochrane Syst Rev [Internet]. 2018, 2(2), CD004876. Available at https://www.cochrane.org/CD004876/ARI_vaccines-preventing-seasonal-influenza-and-its-complications-people-aged-65-or-older
23. Ng QX, Venkatanarayanan N, Ho CYX. Clinical use of Hypericum perforatum (St John's Wort) in depression: A meta-analysis. J Affect Disord. 2017;210:211-21.
24. Linde K, Berner M, Kriston L. St. John's Wort for major depression. Cochrane Syst Rev [Internet]. 2008(4):CD000448. [accessed 5 Aug 2018]; Available at https://www.cochrane.org/CD000448/DEPRESSN_st.-johns-wort-for-treating-depression.
25. Prescrire. Millepertuis et états dépressifs. [St. John's wort and depression]. Rev Prescrire. 2004;24(250):362-9. Available online: https://www.prescrire.org/aLaUne/dossierMillepertuis.php
26. ANSES. Avis de l'Agence nationale de sécurité sanitaire de l'alimentation, de l'environnement et du travail" relatif aux risques liés à la présence de « Levure de riz rouge » dans les compléments alimentaires. [Opinion of the National Agency for Food, Environmental and Occupational Health Safety" relating to the risks associated with the presence of "Red yeast rice" in food supplements]. Maison-Alfort; 2014 p1-34. Report No.: 2012-SA-0228.
27. Klimek M, Wang S, Ogunkanmi A. Safety and Efficacy of Red Yeast Rice (Monascus purpureus) as an Alternative Therapy for Hyperlipidemia. Pharm Ther. 2009;34(6):313-27.
28. Swissmedic. La commercialisation de préparations à base de Monascus purpureus (levure de riz rouge) est illicite en Suisse. [The marketing of preparations based on Monascus purpureus (red yeast rice) is illegal in Switzerland]. Swissmedic; Bern, Switzerland, 2012 p.1-4.
29. Prescrire. Levure de riz rouge. [Red rice yeast]. Rev Prescrire. 2015;35(375):18.
30. Solomon DH. Nonselective NSAIDs : Adverse cardiovascular effects. UpTodate. 2018, 24 March. Available online: https://www.uptodate.com/contents/nsaids-adverse-cardiovascular-effects#H92624237
31. Prescrire. AINS et troubles cardiovasculaires graves : surtout les coxibs et le diclofénac. [NSAIDs and serious cardiovascular disorders: especially coxibs and diclofenac]. Rev Prescrire. 2015;35(384). Available online: https://www.prcrire.org/Fr/3/31/51192/0/NewsDetails.aspx

Table S3: Adherence to EBP according to age band

	Age band				
Adherence to EBP	< 30 yrs (n=193)	30-39 yrs (n=167)	40-49 yrs (n=105)	≥ 50 yrs (n=130)	All (n=595)
EBP-	66 (34.2%)	76 (45.5%)	50 (47.6%)	59 (45.4%)	251 (42.2%)
EBP+	127 (65.8%)	91 (54.5%)	55 (52.4%)	71 (54.6%)	344 (57.8%)
	$p = 0.054$				
